# Calcinosis as a complication of juvenile dermatomyositis (JDM)

**DOI:** 10.1186/1546-0096-9-S1-P55

**Published:** 2011-09-14

**Authors:** Malagón Clara, Yépez Ricardo, Contreras Luz

**Affiliations:** 1 Pediatric Rheumatology fellowship program, Faculty of Medicine, Universidad el Bosque, Bogotá, Colombia

## Background

Calcinosis is a common complication of JDM. It may varies on extension and severity. May cause mechanical and aesthetic complications and predispose to infections.

## Aim

To evaluate the frequency and features of calcinosis in a cohort of patients with JDM and identify if there are risk factors.

## Methods

Retrospective descriptive study in 3 pediatric rheumatology centers in Bogota during a period of 20 years. Two groups were identified according to the presence or absence of clinical and radiological signs of calcinosis after a minimum of 2 years of follow up.

## Results

17/42 developed calcinosis associated with earlier onset of the disease, male predominance and chronic. Calcium deposits were classified as: superficial nodular, deep nodular, mass, linear or mixed deposits and calcinosis universalis. 80% had two or more types of deposits. The anatomical areas more frequently affected were the thighs and forearms. Complications included: drainage, chronic ulcers, mass effect, mechanical blockage of the joints and infections. Severe calcinosis was also associated with significant muscle atrophy loss and lipodystrophy.

**Table:** 

GROUP	With calcinosis		Without calcinosis		Pvalue
Number	17		25		
Sex distribution (Male:female)	1.83:1		1:3.16		0,008
Age of onset (average of years)	6,29(1,5-12)		7,08(2-16)		0,633
Delayed diagnosis (>6months)	6/17	35%	5/25	20%	0,268
Monocyclic	1/17	6%	12/25	48%	0,004
Policiclic	3/17	18%	3/25	12%	0,608
Chronic	14/17	82%	7/25	28%	0,001

## Conclusion

Calcinosis is common and severe of JDM. It leads to various complications and treatment response is poor. Early diagnosis and proper treatment may reduce the frequency of this complication.

